# Dyslipidemia is associated with inflammation and organ involvement in systemic lupus erythematosus

**DOI:** 10.1007/s10067-023-06539-2

**Published:** 2023-02-15

**Authors:** Saisai Huang, Zhuoya Zhang, Yiyuan Cui, Genhong Yao, Xiaolei Ma, Huayong Zhang

**Affiliations:** 1 Department of Rheumatology and Immunology, Nanjing Drum Tower Hospital, The Affiliated Hospital of Nanjing University Medical School, 321 Zhongshan Road, Nanjing, 210008 Jiangsu China; 2 Department of Rheumatology and Immunology, Nanjing Drum Tower Hospital Clinical College of Nanjing University of Chinese Medicine, 321 Zhongshan Road, Nanjing, 210008 Jiangsu China

**Keywords:** Disease activity, Dyslipidemia, Glucocorticoids, Lupus, Renal damage

## Abstract

**Introduction:**

Disturbed lipid metabolism was observed in systemic lupus erythematosus (SLE) patients. This study aimed to evaluate the relationships between dyslipidemia and visceral organ involvement, disease severity, inflammatory factors, and drug intake in SLE patients.

**Method:**

Inpatients with SLE (*n* = 105) and healthy controls (HC) (*n* = 75) were recruited in this study. Clinical and laboratory data were collected from patient records. The concentrations of tumor necrosis factor receptors superfamily member1A (TNFRSF1A), member1B (TNFRSF1B) and adipokine angiopoietin-like 4 (ANGPTL4) in plasma were measured by ELISA.

**Result:**

Compared to HC, serum levels of triglyceride (TG), total cholesterol (TC), low-density lipoprotein (LDL), and apolipoprotein B (ApoB) were significantly increased, while high-density lipoprotein (HDL) and apolipoprotein A1 (ApoA1) were decreased in SLE patients. Patients with higher disease activity and renal damage suffered from more severe dyslipidemia. Renal functional parameters were closely correlated with serum lipid levels. Inflammatory factors were associated with dyslipidemia. The levels of TNFRSF1A and TNFRSF1B were obviously increased and associated with kidney involvement in SLE patients. Patients with high-dose glucocorticoid intake showed more severe dyslipidemia.

**Conclusions:**

Attention should be paid to the dyslipidemia of SLE. Dyslipidemia is associated with inflammation and organ involvement in SLE. These findings might provide a new strategy for the treatment of SLE.

**Table:** 

**Key Points** *• Serum levels of TG, TC, LDL, and ApoB were significantly increased, while HDL and ApoA1 were decreased in SLE patients.* *• Patients with higher disease activity and renal damage suffered from more severe dyslipidemia. Renal functional parameters and inflammatory factors were closely correlated with serum lipid levels.* *• Patients with high-dose glucocorticoid intake showed more severe dyslipidemia.* *• These findings might provide a new strategy for the treatment of SLE.*

**Supplementary Information:**

The online version contains supplementary material available at 10.1007/s10067-023-06539-2.

## Introduction

Systemic lupus erythematosus (SLE) is a common and potentially fatal autoimmune disease characterized by autoantibody-associated multiorgan injuries, including the renal, cardiovascular, neural, and cutaneous systems, primarily affecting women at childbearing age [1]. Dyslipidemia is a lipid-metabolism disorder characterized by increasing or decreasing serum lipid fraction (lipoprotein). It is well established that dyslipidemia is a common feature in SLE patients [2, 3], being a vital risk for heart failure [4], cardiovascular disease (CVD) [5], and kidney disease [6].

Although the pathogenesis of dyslipidemia in SLE has not been clearly identified, many evidences support the hypothesis that several drugs commonly used in SLE patients induce undesirable effects on lipid metabolism, such as corticosteroids [7], cyclosporine A [8], and tacrolimus [9]. On the other hand, immune response and metabolic regulation are highly integrated. Metabolic dysfunction can be triggered by a chronic excess of nutrients like lipids and glucose; these signals also simultaneously trigger inflammatory responses [10]. Thus, the immune disorder may contribute to the abnormal lipid metabolism of SLE.

This study aimed to systemically analyze the lipid profiles according to disease activity, inflammatory factors, visceral organ involvement, and the use of glucocorticoids in SLE. Clarifying the pattern of dyslipidemia would provide useful information for the treatment of SLE patients.

## Methods

### Patients

A total of 105 SLE patients, aged from 13 to 71 years old, who were admitted to the ward of Nanjing Drum Tower Hospital were recruited. All patients fulfilled the SLE diagnostic criteria of the American College of Rheumatology [11]. Seventy-five age and sex-matched healthy controls (HC) were from the medical examination center of our hospital. All the subjects were given informed consent for the collection of peripheral blood. All plasma samples were stored at −80℃ prior to use. The protocol of this study was approved by the Ethics Committee of the institute.

### Clinic and laboratory indexes

Data were collected from the patient records using electronic data processing upon approval. The data collected included demographic information, serum lipids including triglyceride (TG), total cholesterol (TC), high-density lipoprotein (HDL), low-density lipoprotein (LDL), apolipoprotein A1 (ApoA1), apolipoprotein B (ApoB), renal function such as 24-h urine protein, creatinine (Cr), blood urea nitrogen (BUN), uric acid (UA), liver function including alanine transaminase (ALT), aspartate transaminase (AST), total protein (TP), albumin (ALB), globulin (GLO), albumin globulin rate [A/G], immunological indexes including immunoglobulin A, immunoglobulin G, immunoglobulin E, immunoglobulin M, complement component 3, complement component 4, systemic inflammation such as C-reactive protein (CRP), erythrocyte sedimentation rate (ESR). Disease activity was defined according to systemic lupus erythematosus disease activity index (SLEDAI) [12] and 0–4 means inactive (*n*=11), 5–9 means mild (*n*=34), 10–14 means moderate (*n*=38), ≥15 means severe (*n*=22). We divided SLE patients into the lupus nephritis (LN) group (*n*=81) and the non-LN group (*n*=24) according to whether the kidney was involved. The patients were also divided into three subgroups according to daily glucocorticoid dose (prednisone or its equivalent): ≤15mg/day (*n*=41); ≤30mg/day and > 15mg/day (*n*=31); >30mg/day (*n*=33).

### ELISA for ANGPTL4, TNFRSF1A, TNFRSF1B

The plasma levels of ANGPTL4 (Raybiotech, Atlanta, American), TNFRSF1A (FMS, Nanjing, China), and TNFRSF1B (FMS, Nanjing, China) were measured by ELISA kits according to the manufacturer’s instructions.

### Statistical analysis

GraphPad Prism software was used for statistical analyses. Differences in the level of lipids between SLE patients and controls were assessed using *t* test analyses. Pearson correlation was estimated to examine the relationship between all traits. The results are expressed as mean ± SEM (stand error of mean) and *P* < 0.05 was considered significant.

## Results

### Dyslipidemia and dyslipoproteinemia in SLE patients

This study covered a population of 105 SLE patients and 75 HC. Detailed clinical characteristics of SLE patients are shown in Table 1. SLE patients showed a pattern of dyslipidemia and dyslipoproteinemia, characterized with the increase of serum TG, TC, LDL, and ApoB, while the decrease of serum HDL and ApoA1 (Table 2).

**Table 1:** Clinical characteristic of SLE patients

	Patients with SLE (*n*=105)
Baseline characteristics	
Age, year	33.03±1.21
Disease duration, year	6.10±0.62
Gender (female/male)	94/11
SLEDAI score	10.70±0.57
24-h urine protein, mg	3866.00±557.30
Organ involvement	
Renal involvement, *n* (%)	81 (77.14%)
Cutaneous, *n* (%)	48 (45.71%)
Gastrointestinal, *n* (%)	10 (9.52%)
Hematologic, *n* (%)	43 (40.95%)
Neuropsychiatric, *n* (%)	7 (6.67%)
Musculoskeletal, *n* (%)	45 (42.86%)
Comorbidities	
Diabetes, *n* (%)	3 (2.86%)
Hypertension, *n* (%)	44 (41.90%)
Pulmonary arterial hypertension, *n* (%)	15 (14.28%)
Hypothyroidism, *n* (%)	19 (18.10%)
Medication	
Prednisone (mg/d)	27.35±1.93
Hydroxychloroquine, *n* (%)	76 (72.38%)
Tacrolimus, *n* (%)	17 (16.19%)
Mycophenolate Mofetil, *n* (%)	28 (26.67%)
Cyclophosphamide, *n* (%)	32 (30.48%)
Leflunomide, *n* (%)	15 (14.28%)
Thalidomide, *n* (%)	6 (5.71%)
Azathioprine, *n* (%)	4 (3.8%)

*SLE*, systemic lupus erythematosus; *SLEDAI*, systemic lupus erythematosus disease activity index

**Table 2:** Comparison of serum lipids and inflammatory cytokines between SLE patients and HC

	HC	SLE patients	*P* value
TG (mmol/l)	0.93±0.03	1.97±0.12	<0.0001*
TC (mmol/l)	4.27±0.07	5.24±0.17	<0.0001*
HDL (mmol/l)	1.43±0.04	1.24±0.05	0.003*
LDL (mmol/l)	2.16±0.05	2.99±0.14	<0.0001*
ApoA1 (g/l)	1.37±0.03	1.21±0.04	0.003*
ApoB (g/l)	0.83±0.02	1.06±0.04	<0.0001*
TNFRSF1A (ng/ml)	0.47±0.03	2.00±0.21	<0.0001*
TNFRSF1B (ng/ml)	2.38±0.15	10.86±1.03	<0.0001*
ANGPTL4 (pg/ml)	1509±371.2	1244±471.3	0.76

*SLE*, systemic lupus erythematosus; *HC*, healthy control; *TG*, triglyceride; *TC*, cholesterol; *HDL*, high-density lipoprotein; *LDL*, low-density lipoprotein; *ApoA1*, apolipoprotein A1; *ApoB*, apolipoprotein B; *TNFRSF1A*, tumor necrosis factor receptors superfamily, member1A; *TNFRSF1B*, tumor necrosis factor receptors superfamily, member1B; *ANGPTL4*, adipokine angiopoietin-like 4

*P<0.05

### Lipid profiles were associated with liver and renal dysfunction in SLE patients

As we know, liver is one of the most important organs playing a vital role in lipid metabolism [13]. As shown in Table 3, the relationship between lipid profiles and liver parameters was evaluated. We found that TP and ALB were negatively correlated with TG, TC, LDL, LDL/HDL, and ApoB, while ALT and AST had no correlation with serum lipid profiles. The GLO was negatively correlated with TC, LDL. A/G showed a negative correlation with TG as well as LDL/HDL and positively correlated with HDL. We also found that immunoglobulin G was negatively correlated with TC, HDL, LDL, ApoA1, and ApoB, while immunoglobulin A, immunoglobulin M, immunoglobulin E, complement component 3, and complement component 4 had no correlation with serum lipid levels (Table 3).

**Table 3:** Correlation between serum lipid levels and clinical index

	TG		TC		HDL		LDL		LDL/HDL		ApoA1		ApoB	
	*r*	*P*	*r*	*P*	*r*	*P*	*r*	*P*	*r*	*P*	*r*	*P*	*r*	*P*
ALT	0.11	0.29	−0.13	0.19	−0.11	0.28	−0.15	0.12	−0.04	0.64	−0.02	0.81	−0.08	0.41
AST	0.04	0.67	−0.10	0.30	−0.12	0.22	−0.15	0.13	−0.07	0.48	−0.04	0.66	−0.03	0.74
TP	−0.26	0.03*	−0.41	0.0007*	−0.05	0.74	−0.41	0.0007*	−0.26	0.006*	0.07	0.59	−0.38	0.001*
ALB	−0.41	0.0006*	−0.46	0.0001*	0.24	0.06	−0.44	0.0003*	−0.43	<0.0001*	0.20	0.11	−0.51	<0.0001*
GLO	0.01	0.93	−0.28	0.02*	−0.14	0.26	−0.33	0.007*	−0.02	0.82	−0.06	0.65	−0.20	0.11
A/G	−0.27	0.03*	−0.01	0.95	0.27	0.03*	0.04	0.73	−0.21	0.03*	0.16	0.19	−0.11	0.39
24-h urine protein	0.40	<0.0001*	0.62	<0.0001*	−0.009	0.93	0.61	<0.0001*	−0.07	0.51	−0.001	0.99	0.66	<0.0001*
Cr	0.04	0.66	0.33	0.0006*	0.24	0.05	0.26	0.008*	−0.07	0.48	0.17	0.09	0.24	0.01*
BUN	0.15	0.14	0.26	0.007*	0.005	0.96	0.20	0.04*	0.03	0.73	−0.001	0.99	0.27	0.006*
UA	0.12	0.24	0.29	0.02*	0.07	0.48	0.26	0.007*	0.11	0.26	0.06	0.56	0.27	0.006*
IgA	0.02	0.86	−0.19	0.06	−0.22	0.06	−0.16	0.12	0.11	0.33	−0.20	0.06	−0.07	0.51
IgG	−0.11	0.29	−0.40	<0.0001*	−0.34	0.001*	−0.38	<0.0001*	0.11	0.29	−0.29	0.005*	−0.26	0.03*
IgM	0.03	0.77	−0.05	0.66	−0.13	0.22	−0.04	0.71	0.10	0.33	−0.05	0.64	0.03	0.77
IgE	0.03	0.81	0.08	0.47	−0.16	0.14	0.21	0.06	0.08	0.44	−0.21	0.06	0.16	0.13
C3	−0.17	0.10	−0.01	0.91	0.13	0.23	0.01	0.97	−0.03	0.71	0.20	0.05	−0.09	0.38
C4	−0.06	0.56	0.05	0.62	0.03	0.75	0.03	0.77	−0.01	0.96	0.08	0.47	0.02	0.82
ESR	0.26	0.01*	0.20	0.05	−0.29	0.005*	0.20	0.05	0.32	0.001*	−0.20	0.04*	0.32	0.002*
CRP	0.15	0.13	−0.26	0.05	−0.43	<0.0001*	−0.13	0.19	0.08	0.41	−0.42	<0.0001*	−0.0006	0.99
ANGPTL4	0.09	0.47	0.03	0.80	−0.38	0.003*	0.07	0.60	0.27	0.03*	−0.30	0.02*	0.15	0.25
TNFSF1A	0.40	0.002*	0.19	0.13	−0.13	0.33	0.20	0.13	0.18	0.17	−0.12	0.36	0.21	0.11
TNFSF1B	0.35	0.006*	0.12	0.38	−0.18	0.18	0.11	0.41	0.32	0.01*	−0.18	0.16	0.15	0.27

*Cr*, creatinine; *BUN*, blood urea nitrogen; *UA*, uric acid; *ALT*, alanine transaminase; *AST*, aspartate transaminase; *TP*, total protein; *ALB*, albumin; *GLO*, globulin; *A/G*, albumin globulin rate; *IgA*, immunoglobulin A; *IgG*, immunoglobulin G; *IgM*, immunoglobulin M; *IgE*, immunoglobulin E; *C3*, complement component 3; *C4*, complement component 4; *CRP*, C-reactive protein, *ESR*, erythrocyte sedimentation rate;*ANGPTL4*, adipokine angiopoietin-like 4; *TNFRSF1A*, tumor necrosis factor receptors superfamily, member1A; *TNFRSF1B*, tumor necrosis factor receptors superfamily, member1B

*P<0.05，Bolded italics represent significant values

**Fig. 1:**
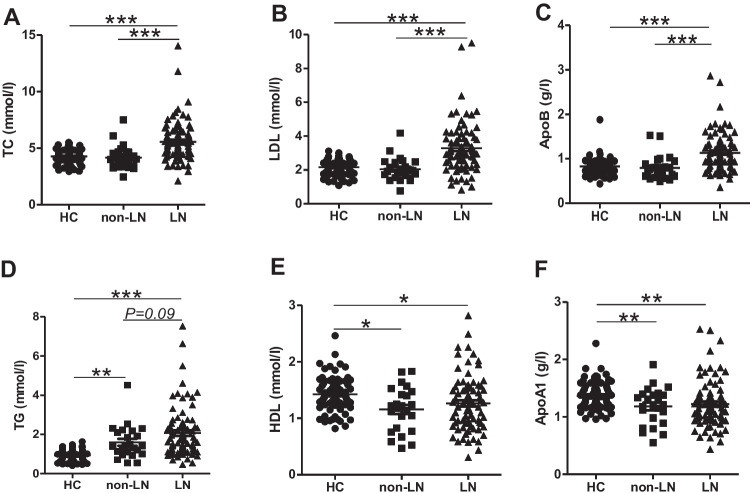
Serum lipid levels in LN and non-LN patients. LN patients had higher levels of TC (**A**), LDL (**B**), and ApoB (**C**) compared to non-LN patients. The levels of TG (**D**), HDL (**E**), and ApoA1 (**F**) were comparable between LN and non-LN groups. LN, lupus nephritis. LN, *n*=81, non-LN, *n*=24. *P<0.05, **P<0.01, ***P<0.001

Since the kidney also takes part in lipid metabolism [14] and is the most commonly involved organ in SLE [15], we next explored the relationship between renal function and serum lipid parameters. As shown in Fig. 1, LN patients had much higher TC, LDL, and ApoB compared to patients without LN. The level of 24-h urine protein showed a positive correlation with serum TG, TC, LDL, and ApoB. The Cr and BUN as well as UA were positively correlated with TC, LDL, and ApoB (Table 3).

### Hyperlipidemia was related to more severe disease activity and inflammation in SLE patients

**Fig. 2:**
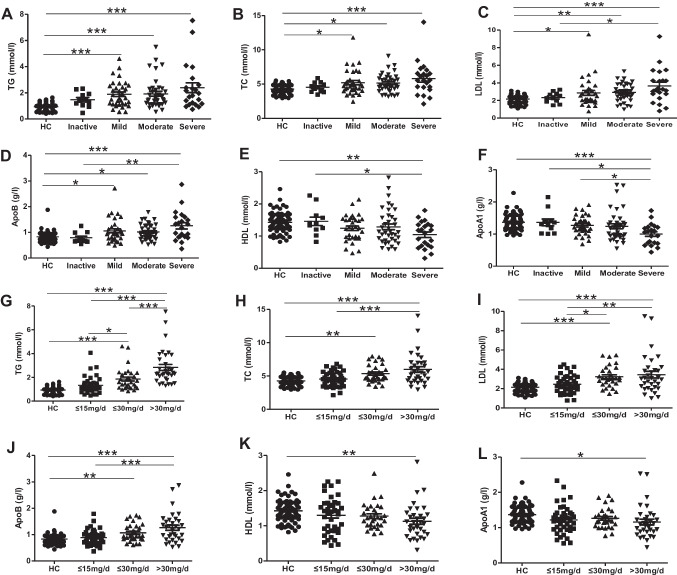
Comparison of serum lipid parameters in SLE patients with different disease activity and glucocorticoid intake. The patients with severe disease activity had higher levels of LDL (**C**), ApoB (**D**), and lower levels of HDL (**E**) and ApoA1 (**F**). The levels of TG (**A**) and TC (**B**) were comparable in SLE patients with different disease activities. inactive, *n*=11, with SLEDAI score 0–4, mild, *n*=34, with SLEDAI score 5–9, moderate, *n*=38, with SLEDAI score 10–14, severe, *n*=22, with SLEDAI score ≥15. Patients with high-dose of prednisone (>30mg/day) showed higher serum TG (***G***), TC (***H***), LDL (***I***), ApoB (***J***), and lower HDL (***K***), and ApoA1 (**L**). HC, *n*=75, ≤15mg/day, *n*=41, ≤30mg/day, *n*=31, >30mg/day, *n*=33. * *P*＜0.05,** *P*＜0.01,*** *P*＜0.001

As shown in Fig. 2, patients with severe disease activity showed higher levels of serum LDL, ApoB, and lower levels of serum HDL, ApoA1 than other groups.

It is reported that inflammation, a hallmark of SLE, regulates the lipolysis process through inhibiting lipoprotein lipase (LPL) activity [16]. We next tried to explore whether there was a correlation between the inflammatory status and lipid profiles in SLE patients. We found that ESR was positively associated with TG, LDL/HDL, and ApoB and negatively associated with HDL and ApoA1, while CRP was negatively correlated with HDL and ApoA1 (Table 3).

*P<0.05, **P<0.01, ***P<0.001

### Dyslipidemia may participate in kidney injury in SLE patients via tumor necrosis factor receptors

To further explore the relationships of dyslipidemia, inflammatory cytokines, and kidney injury in SLE, plasma levels of inflammatory factors ANGPTL4, TNFSF1A, and TNFSF1B were examined. Compared with HC, both the concentrations of TNFSF1A and TNFSF1B were significantly increased in SLE patients, while the level of ANGPTL4 was comparable between the two groups (Table 2). In addition, a significant negative correlation was observed between ANGPTL4 level and HDL as well as ApoA1, while plasma TNFSF1A and TNFSF1B were both positively correlated with TG level (Table 3). More importantly, we found the levels of TNFSF1A and TNFSF1B were positively correlated with 24-h urine protein and BUN as well as Cr (Table 4). As dyslipidemia was also correlated with renal dysfunction, we supposed that hyperlipidemia in SLE patients might participate in kidney injury through TNFSF1A and TNFSF1B.

**Table 4:** The levels of tumor necrosis factor receptors were correlated with renal functions in SLE patients

	24-h urine protein		Cr		BUN		UA	
	*r*	*P*	*r*	*P*	*r*	*P*	*r*	*P*
ANGPTL4	0.12	0.41	0.11	0.7	0.29	0.03*	0.10	0.45
TNFSF1A	0.37	0.007*	0.64	0.001*	0.57	0.001*	0.24	0.07
TNFSF1B	0.34	0.01*	0.43	0.001*	0.38	0.003*	0.10	0.47

*ANGPTL4*, adipokine angiopoietin-like 4; *TNFRSF1A*, tumor necrosis factor receptors superfamily, member1A; *TNFRSF1B*, tumor necrosis factor receptors superfamily, member1B; *Cr*, creatinine, *BUN*, blood urea nitrogen; *UA*, uric acid

*P<0.05

### Glucocorticoids were associated with hyperlipidemia in SLE

Since drugs may influence serum lipid profiles, we next analyzed serum lipid levels according to the doses of prednisone in SLE patients. All 105 patients were treated with glucocorticoids. According to the different drug doses, we divided them into three groups. Figure 2 shows that patients with ≥30mg/day prednisone had higher serum levels of TG, TC, LDL, and ApoB, but lower HDL as well as ApoA1 compared to HC or patients with ≤15mg/day prednisone. These results indicated that the dose of glucocorticoids could influence serum lipid levels in SLE patients.

## Discussion

Changes in lipid metabolism are the most important biomarkers and risks of CVD, which is one of the leading causes of SLE mortality [17]. The reason for the abnormal lipid metabolism in SLE and its influence on lupus is still debatable. In order to further understand of dyslipidemia in SLE, we analyzed the relationship between their lipid profiles and clinical and laboratory parameters in 105 SLE patients. Compared to HC, serum lipids in SLE patients were disordered with increasing TG, TC, LDL, and decreasing HDL. Serum levels of HDL, LDL, ApoA1, and ApoB were significantly correlated with the SLEDAI score. These findings indicate that blood lipid levels reflect disease activities in SLE patients.

As a key organ for energy and nutrient homeostasis, the liver plays a wide range of functions in lipid metabolism including lipogenesis, fatty acid (FA) oxidation, ketogenesis, and lipoprotein secretion [18]. Hepatic involvement in SLE could be due to various factors such as drugs, steatosis, viral hepatitis, vascular thrombosis, and overlaps with autoimmune hepatitis (AIH) or due to SLE itself [19]. Significant negative correlations were found between serum TG, TC, and serum TP, ALB of SLE in our study.

It is widely accepted that renal injury could disturb lipid profiles. Now there are mounting evidences showing that dyslipidemia can, in turn, accelerate renal damage [20]. Around 40–90% patients with SLE have kidney involvement. We found that dyslipidemia and dyslipoproteinemia in SLE patients were obviously associated with renal injury. The patients with severe kidney injury had much more serious abnormality of serum lipid profiles, suggesting that control of lipid profiles might be beneficial for the prevention and treatment of renal damage in lupus patients.

Both clinical observations and basic researches suggest that there is a potential link between inflammation and lipid metabolism [21]. In our study, both ESR and CRP were associated with HDL and ApoA1, but it was reported that there was no association between CRP and dyslipidemia in India SLE patient [22]. These opposite results may be due to the different populations and numbers of patients in two different studies. It is known that tumor necrosis factor alpha (TNFα) can regulate lipid metabolism [23], but the influence of its receptor TNFSF1A and TNFSF1B on serum lipids is not fully understood. ANGPTL4 is a molecular linker between insulin resistance and rheumatoid arthritis [24] and can inhibit LPL activity [25]. In multivariate regression analysis, TG, HDL, and ApoB were the independent predictors of TNFRSF1A and TNFRSF1B. ApoA1 was also an independent predictor of TNFRSF1B. Dyslipidemia was not an independent factor associated with ANGPTL4, CRP, and ESR (additional file: Table S1). However, there is no report on ANGPTL4 level in SLE and its specific role in lupus dyslipidemia remains unknown. In our study, we found all these three inflammatory cytokines had a significant correlation with lipid profile in SLE patients, providing new hints to investigate the pathogenesis of dyslipidemia in SLE patients.

It was reported that the TNF receptor was associated with a decline in kidney function in patients with stable ischemic heart disease and diabetes [26, 27]. In our study, we found the levels of TNFSF1A and TNFSF1B were significantly correlated with kidney function in SLE patients. Our results also showed the patients with severe kidney injury had much more serious abnormal serum lipid profiles, which suggested hyperlipidemia may participate in kidney involvement in SLE patients through TNFα receptors.

In consideration of the possible influence of drugs on serum lipids, we analyzed the use of prednisone in SLE patients. In our study, we found that prednisone had deleterious effects on serum lipids in a dose-dependent manner, which may be due to that glucocorticoid can increase lipolysis, decrease glucose uptake, and release free fatty acids into circulation [28].

The limitations of this study include the following: First, given the small number of cases, we could not perform comprehensive validation studies. Further studies in a larger population are needed. Second, the current research targeted a cross-sectional study and did not include following-up data. Finally, this was a single-center study, and some multi-center studies are still needed to validate and further explore.

## Conclusion

Our findings suggested that SLE patients had a lipid profile abnormality which was associated with organ involvement, inflammation, and prednisone. Abnormal lipid metabolism had been strongly implicated as a key mediator of progressive renal injury and immune disorder, which could provide a new strategy for the treatment of SLE.

## Supplementary informations

Below is the link to the electronic supplementary material.

Supplementary 1 (DOCX 13.7 KB)

## Data Availability

The data underlying this article are available in the article.
