# Supplementary material for: Dyslipidemia is associated with inflammation and organ involvement in systemic lupus erythematosus
Source: Clin Rheumatol. 2023 Feb 15;42(6):1565–72. doi: 10.1007/s10067-023-06539-2 (PMC10203001; doi:10.1007/s10067-023-06539-2)
Supplement: Supplementary file 1 — Supplementary 1 (DOCX 13.7 KB) [file 10067_2023_6539_MOESM1_ESM.docx]

Supplementary Table 1. Multivariate regression analysis of biomarker prediction of clinical disease

|  | Independent factor of ANGPTL4 | Independent factor of TNFSF1A | Independent factor of TNFSF1B | Independent factor of CRP | Independent factor of ESR |
| --- | --- | --- | --- | --- | --- |
| TG | NS | 0.016 | 0.001 | NS | NS |
| TC | NS | NS | NS | NS | NS |
| HDL | NS | 0.022 | 0.004 | NS | NS |
| LDL | NS | NS | NS | NS | NS |
| ApoA1 | NS | NS | 0.004 | NS | NS |
| ApoB | NS | 0.015 | 0.003 | NS | NS |
